# Anodal Transcranial Direct Current Stimulation Enhances Short-Term Balance During Locomotive Training in Older Adults with Locomotive Syndrome: A Pilot Randomized Controlled Trial

**DOI:** 10.3390/geriatrics11030056

**Published:** 2026-05-07

**Authors:** Hitoshi Shitara, Tsuyoshi Tajika, Eiji Takasawa, Hirotaka Chikuda

**Affiliations:** Department of Orthopedic Surgery, Graduate School of Medicine, Gunma University, 3-39-22 Showa-Machi, Maebashi 371-8511, Japan; tajika@gunma-u.ac.jp (T.T.); e-takasawa@gunma-u.ac.jp (E.T.); chikuda-tky@umin.ac.jp (H.C.)

**Keywords:** transcranial direct current stimulation, locomotive syndrome, balance, older adults, motor cortex, postural control, randomized controlled trial

## Abstract

**Background/Objectives**: Locomotive syndrome (LS) is associated with impaired balance and functional decline in older adults. Although locomotive training (LT) improves mobility, whether central neuromodulation enhances short-term balance adaptation remains unclear. This pilot randomized controlled trial examined the additive effect of anodal transcranial direct current stimulation (tDCS) with LT on balance. **Methods**: Sixteen community-dwelling adults aged ≥ 65 years with LS were randomized (1:1:1) to anodal tDCS + LT (*n* = 6), sham tDCS + LT (LT group, *n* = 6), or anodal tDCS alone (*n* = 4). Participants underwent five consecutive days of intervention. The primary outcome was eyes-open single-leg stance time, assessed before stimulation and at 10, 20, 50, and 80 min during and after stimulation on days 1–5. Group × time interactions were evaluated using linear mixed-effects models adjusted for baseline and age. Long-term outcomes were assessed on days 1, 5, and 12. **Results**: In the primary analysis, a significant group × time interaction for right-sided single-leg stance time was observed between the anodal tDCS + LT and the LT groups (F(1,58) = 6.08, *p* = 0.017; β = 0.966), indicating greater within-day improvement with combined therapy, but not in sensitivity analyses treating time as a categorical variable. No significant interactions were observed on the left side. Secondary outcomes showed time-dependent improvements without consistent group-specific effects or significant group × day interactions over the long term. No serious adverse events occurred. **Conclusions**: Anodal tDCS with LT improved short-term balance in the primary analysis; however, these effects were model-sensitive and not sustained over 12 days. These findings should be considered preliminary and hypothesis-generating. Larger trials are needed to determine optimal stimulation dosing and long-term efficacy.

## 1. Introduction

Locomotive syndrome (LS) is characterized by impaired mobility because of musculoskeletal dysfunction and is associated with increased risk of disability and need for long-term care in older adults [1]. Declines in balance and lower extremity function are central features of this condition and substantially contribute to falls, functional dependence, and reduced quality of life [2]. Notably, even in early-stage LS, impairments in postural control may emerge before overt muscle loss or sarcopenia becomes clinically apparent [3,4,5].

Aging involves a gradual decline in skeletal muscle mass and strength, a condition known as sarcopenia [5,6]. In older adults, decreases in motor unit number, neural activation, and intrinsic muscle contractility lead to reduced maximal force generation [7]. Consequently, short-term neuromodulatory interventions may have limited capacity to immediately increase peripheral muscle strength in this population. In contrast, postural control relies primarily on central motor planning, sensorimotor integration, and swift neural adaptations [8]. Since balance performance depends more on cortical and subcortical processing than on muscle hypertrophy alone, central neural-targeted interventions may more effectively improve balance outcomes in the short term.

Transcranial direct current stimulation (tDCS) is a noninvasive neuromodulatory technique that modulates cortical excitability by applying low-intensity electrical currents to targeted brain regions [9,10]. Anodal stimulation of the primary motor cortex (M1) enhances corticospinal excitability and facilitates motor learning and task performance [9,10]. Previous studies have demonstrated beneficial effects of tDCS on motor recovery, balance, and gait in neurological populations, such as those with stroke and Parkinson’s disease [11,12,13]. However, evidence of its additive value in community-dwelling older adults with musculoskeletal mobility impairment remains limited.

Locomotive training (LT), a structured program targeting balance and lower extremity function, is widely implemented to prevent functional decline in older adults [1,4]. Although LT improves mobility and postural stability [14], it remains unclear whether its effects are enhanced by central neuromodulation. Given age-related reductions in peripheral muscle capacity and the central dependence of postural control, we hypothesized that anodal tDCS would preferentially enhance short-term balance adaptation rather than produce immediate increases in isolated muscle strength.

This pilot randomized controlled trial examined whether five consecutive sessions of anodal tDCS enhances the effects of LT in older adults with LS. We hypothesized that (1) anodal tDCS combined with LT would increase the rate of within-day improvement in balance performance, as reflected by single-leg stance time, compared with LT alone; (2) this additive effect would be more pronounced in postural control than in isolated muscle strength measures; and (3) repeated sessions might produce cumulative functional benefits, although sustained between-group differences would depend on stimulation dose and exposure duration.

## 2. Materials and Methods

### 2.1. Participants

Participants, aged 65 years or older and screening positive on the Loco-Check questionnaire [15,16], were recruited from the local community surrounding Gunma University Hospital in Maebashi, Gunma, Japan, via posters and flyers.

Demographic characteristics, including age, sex, and height, were recorded at baseline. Body composition was assessed using a multi-frequency segmental body composition analyzer (MC-780A; Tanita, Tokyo, Japan). Measurements included body weight (kg), body mass index (BMI; kg/m^2^), fat mass (kg), fat-free mass (kg), total body fat percentage (%), and total muscle mass (kg).

Sarcopenia was defined according to the Asian Working Group for Sarcopenia 2025 criteria [6].

### 2.2. Trial Design

This was a pilot randomized controlled trial with three parallel arms. The primary comparison, between anodal tDCS plus LT (tDCS + LT) [16] and sham tDCS + LT (LT) groups, used a double-blind design to assess the additive effects of anodal tDCS.

Participants were randomly allocated (1:1:1) to tDCS + LT, LT, or anodal tDCS alone (tDCS group). Blinding was applied to the anodal versus sham tDCS groups combined with LT. The tDCS group served as an exploratory reference to evaluate tDCS effects without LT; no sham LT was used.

### 2.3. Changes to Trial Protocol

The original protocol planned to enroll 15 participants per group. However, because of challenges in recruiting older adults for a 12-day intervention with repeated assessments, enrollment was slower than expected. Recruitment ceased after enrolling 16 participants, yielding a smaller-than-planned sample size.

### 2.4. Trial Setting

All interventions and assessments were conducted in a research setting at Gunma University Hospital in Maebashi, Gunma, Japan. During balance assessments and LT, participants were closely supervised by an experienced orthopedic surgeon, who remained beside the participant to provide immediate physical support in case of instability or loss of balance. No safety harness or rail system was used.

### 2.5. Eligibility Criteria

Inclusion criteria were age ≥65 years, absence of joint pain affecting activities of daily living, and presence of LS, defined as at least one positive response on the Loco-Check questionnaire [15,16].

LS was screened using the Loco-Check questionnaire, a validated 7-item tool assessing locomotive function based on activities of daily living. The items included inability to put on socks while standing on one leg, frequent stumbling or slipping at home, need for a handrail when climbing stairs, difficulty performing physically demanding housework, difficulty walking home while carrying a shopping bag weighing approximately 2 kg, difficulty walking continuously for 15 min, and inability to cross a road at a pedestrian crossing before the traffic light changes.

Exclusion criteria included contraindications to tDCS, such as implanted medical devices, metallic implants near the head or neck, history of epilepsy, active skin inflammation or open wounds at electrode placement sites, pregnancy, or diagnosis of neurological or psychiatric disorders.

### 2.6. Intervention and Comparator

**Figure 1:**
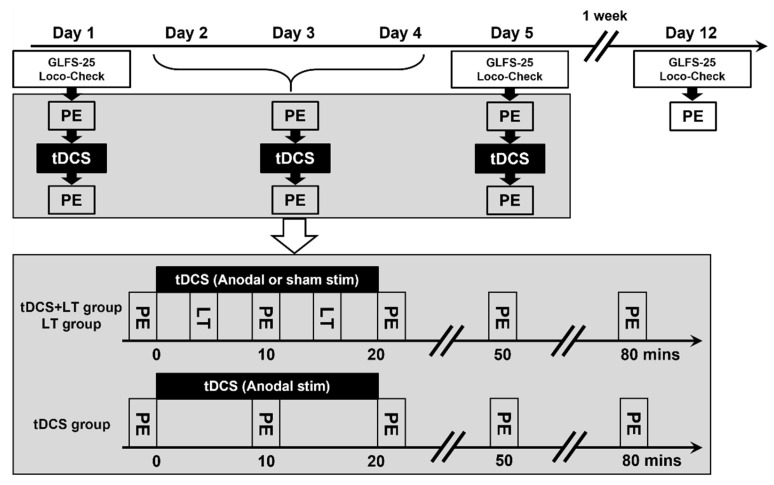
Study protocol. Physical examinations—including handgrip, knee extension, finger-pinch, and toe-pinch strengths; stand-up test; two-step test; single-leg standing time with eyes open; and Timed Up-and-Go test—were performed on days 1–5 and 12. Anodal or sham transcranial direct current stimulation (tDCS) was administered on days 1–5. In the anodal tDCS + LT and sham tDCS + LT groups, locomotive training (LT) consisted of single-leg standing with eyes open and squatting. The GLFS-25 and Loco-Check questionnaires were administered on days 1, 5, and 12. PE, physical examination; LT, locomotive training; LT group, sham tDCS + LT group; tDCS group, anodal tDCS alone group; GLFS-25, 25-question Geriatric Locomotive Function Scale.

Interventions were administered for 5 consecutive days (days 1–5). Participants received tDCS, LT, or tDCS + LT, depending on group assignment (Figure 1).

#### 2.6.1. Transcranial Direct Current Stimulation

Direct current stimulation was delivered using a DC Stimulator Plus (NeuroConn, Ilmenau, Germany) through two saline-soaked sponge surface electrodes (surface area: 35 cm^2^ each) soaked in a 15 mM NaCl solution. The anodal electrode was positioned over the bilateral lower limb representation of M1, identified using Cz as a reference point according to the international 10–20 EEG system. A cathodal electrode was placed over the contralateral supraorbital region.

Anodal tDCS was delivered at an intensity of 2 mA for a total duration of 20 min, including a 15 s ramp-up and 15 s ramp-down period.

For sham stimulation, the electrode montage and setup were identical; however, current was applied for only 30 s and then discontinued, providing the initial sensation of stimulation without inducing lasting neuromodulatory effects.

#### 2.6.2. Locomotion Training

LT [16] was performed concurrently with tDCS and included two exercise sessions per day: one during the first 10 min of stimulation and the other during the subsequent 10 min. The LT program included eyes-open single-leg standing and squatting, both of which are recommended components of LS prevention programs.

Eyes-open single-leg standing [16]: Participants lifted one lower limb slightly off the floor and maintained a single-leg stance with eyes open. Each leg was trained for up to 1 min per session. If participants were unable to maintain the position continuously, they were allowed to perform multiple shorter attempts within the 1 min period.

Squatting [16]: Participants slowly flexed their knees and returned to an upright position, completing five repetitions per session. For participants unable to perform standard squats, a modified chair-rise exercise with hand support was substituted.

Participants assigned to the anodal tDCS-only group received tDCS without concomitant LT. All interventions were supervised by an orthopedic surgeon with more than 20 years of clinical experience (T.T.), and adherence to the intervention protocol was monitored throughout the study. The supervising surgeon was not involved in outcome assessments.

### 2.7. Outcomes

#### 2.7.1. Primary Outcome

The primary outcome was single-leg stance time with eyes open [17], measured for the right and left legs. For the primary analysis, we prespecified the group × time interaction as the main test to evaluate the additive effect of anodal tDCS combined with LT on within-day changes in repeated measurements. All outcome assessments were performed by orthopedic surgeons with more than 10 years of clinical experience (E.T. and H.S.), who were blinded to group allocation.

Single-leg stance time with eyes open: Static balance was assessed using a single-leg stance test with eyes open, which has demonstrated excellent test–retest reliability and discriminant validity in older adults [17]. Participants were instructed to stand barefoot on their preferred leg for as long as possible with eyes open while keeping their arms at their sides. Timing began when the non-weight-bearing foot was lifted from the floor and ended when the lifted foot touched the ground, contacted the supporting leg, or when any hand support was used.

#### 2.7.2. Secondary Outcomes

Secondary outcomes included bilateral handgrip strength, finger-pinch strength, knee extension strength, toe-pinch strength, stand-up test score, two-step value, and Timed Up-and-Go (TUG) test performance. These outcomes were assessed before stimulation and at 10, 20, 50, and 80 min during and after stimulation on days 1 through 5, unless otherwise specified. Additionally, LS stage [16], Loco-Check score [16], and the 25-question Geriatric Locomotive Function Scale (GLFS-25) [16] were evaluated before stimulation on days 1, 5, and 12.

Strength measurements: Bilateral handgrip, finger-pinch, knee extension, and toe-pinch strengths were measured twice; the average of the two trials was used for analysis. The intra-rater validity and reliability of these muscle strength measurements have been established in previous studies [18,19,20,21,22].

Grip and pinch strength measurements: Grip strength was assessed using a digital dynamometer (Takei Scientific Instruments Co., Tokyo, Japan) following the standardized positioning protocol recommended by the American Society of Hand Therapists [23]. Participants were seated with the shoulder in adduction and neutral rotation, the elbow flexed at 90°, and the forearm in a neutral position. Key pinch strength was measured using a pinch gauge (MG-4320NC; B&L Engineering, Santa Ana, CA, USA) [23], with participants positioned such that the shoulder, elbow, forearm, and wrist were neutral, and the thumb pad opposed the lateral aspect of the middle phalanx of the index finger.

Knee extension isometric strength measurements: Isometric knee extension strength was assessed using a handheld dynamometer (MicroFET2™; Hoggan Health Industries, Salt Lake City, UT, USA) [22]. Participants were seated with hips and knees flexed at 90°. The dynamometer was positioned on the distal anterior aspect of the leg, against which participants performed maximal isometric knee extension.

Toe-pinch strength measurement: Toe-pinch strength was measured using a toe-gap force gauge (Checkerkun; NI Industrial Co., Ltd., Ageo, Japan) [24,25]. The device consists of a foot stand with a fixed measuring component, a movable measuring component, and a display unit. Participants firmly compressed the fixed and movable measuring components between the first and second toes to quantify inter-toe pressure, which was displayed on the unit.The LS risk test was performed to detect mobility decline, which is strongly associated with disability [26]. It includes two functional assessments—the stand-up test [16] and two-step test [4]—and a self-administered questionnaire [27]. Standardized reference values for these measures were derived from epidemiological studies in the Japanese population [28,29].

The stand-up test evaluated lower-limb muscle strength [16]. Participants attempted to stand from stools at heights of 40 cm (easiest), 30 cm, and 20 cm (most difficult) using both legs, followed by a single-leg stand from a 40 cm stool. Tasks progressed from easiest to most challenging. Success required rising and maintaining posture for 3 s; scores were based on the most difficult task completed.

The two-step test measured maximum stride length [4]. The distance covered by two steps was divided by the participant’s height to yield the two-step value.

The GLFS-25 is a self-reported questionnaire comprising 25 items [27], including 4 on pain and 21 on daily activities over the past month. Items are scored on a 5-point scale (0–4), yielding a total score from 0 to 100.

LS severity was classified into three stages using the LS risk test [29]. Stage 1 indicates early mobility decline, defined by at least one of the following: inability to stand on one leg from a 40 cm stool, two-step value < 1.3, or GLFS-25 score ≥ 7. Stage 2 indicates progressive decline, defined by inability to stand on both legs from a 20 cm stool, two-step value < 1.1, or GLFS-25 score ≥ 16. Stage 3 indicates severe impairment, defined by inability to stand on both legs from a 30 cm stool, two-step value < 0.9, or GLFS-25 score ≥ 24.

The TUG test was administered twice; the shorter time was used for analysis. Participants stood from a 40 cm-high chair, walked 3 m, turned, returned, and sat down. Timing was recorded with a stopwatch. Instructions were: “Walk to the line, turn around, return, and sit down as quickly as possible.” The TUG test has demonstrated excellent test–retest reliability and validity in older adults [30,31].

### 2.8. Harms

Adverse events were monitored throughout the study. Participants receiving tDCS were screened for common side effects, including skin redness, itching, burning at electrode sites, mild headache, and fatigue, via questioning and examination at each session.

Safety during LT was assessed by recording falls or near-falls during sessions.

### 2.9. Sample Size

The primary outcome was eyes-open single-leg stance time. Because no prior studies have evaluated the combined effects of anodal tDCS and LT on this outcome in older adults with LS, an expected effect size could not be determined, precluding a formal a priori sample size calculation. Therefore, this study was designed as a pilot randomized study to assess feasibility and to generate preliminary estimates of effect size and variability to inform the design of future adequately powered confirmatory trials. Based on feasibility considerations and prior pilot studies of tDCS in older adults, we planned to recruit 15 participants per group. However, recruitment proved challenging because of the intensive protocol requiring repeated assessments over 12 days, and the study was completed with a total of 16 participants.

Given the limited sample size, the study was not powered for definitive hypothesis testing, and all findings should be interpreted as pilot and hypothesis-generating.

### 2.10. Randomization

We performed randomization using a computer-generated sequence via the RAND function in Microsoft Excel (Microsoft, Redmond, WA, USA). Participants received anonymous identification numbers and were allocated in a 1:1:1 ratio to the anodal tDCS + LT, sham tDCS + LT, or anodal tDCS-only groups.

An investigator uninvolved in enrollment or assessment generated the sequence.

### 2.11. Allocation Concealment Mechanism

We ensured allocation concealment by restricting access to the randomization list until after participants provided written informed consent and group assignment.

### 2.12. Implementation

Study personnel without access to the randomization sequence enrolled participants. A separate investigator assigned groups using the pre-generated list after obtaining written informed consent. Enrollment and assessment personnel remained blinded to the sequence throughout the study.

### 2.13. Blinding

Post-randomization, participants, outcome assessors, and data analysts were blinded to group allocations. Two assessors (E.T. and H.S.), unaware of the assigned interventions, performed outcome assessments. Investigator H.S., blinded to group identities, conducted data analysis. Only investigator T.T., responsible for intervention supervision, knew the allocations.

Blinding used identical procedures for anodal and sham tDCS, including electrode placement, stimulation duration, and device settings. Assessors were absent from intervention sessions and lacked access to the randomization list. The data analyst received a de-identified dataset with masked group labels until analysis completion.

### 2.14. Statistical Methods

We conducted all analyses according to a prespecified plan and reported them in accordance with the CONSORT statement. We summarized continuous variables as means (SDs), as appropriate. All statistical tests were two-sided with a significance level of α = 0.05.

To assess baseline comparability, we compared demographic and clinical characteristics (age, sex, height, body weight, body mass index [BMI], LS stage, Loco-Check score, and 25-question GLFS-25) among the three groups. We used one-way analysis of variance for continuous variables and Fisher’s exact test for categorical variables.

### 2.15. Within-Day Effects (Days 1–5)

#### 2.15.1. Primary Outcome

The primary outcome was single-leg stance time with eyes open. To assess the additive effect of anodal tDCS combined with LT, we applied linear mixed-effects models (LMMs) to repeated measurements obtained before stimulation and at 10, 20, 50, and 80 min post-stimulation on days 1–5. In this model, repeated measurements obtained across days 1–5 were analyzed jointly in a single model. Therefore, the estimated group × time interaction represents an averaged within-day trajectory across all intervention days rather than day-specific effects. This approach was selected to improve statistical efficiency and model stability given the limited sample size.

For the primary analysis, we included only the sham tDCS + LT and anodal tDCS + LT groups because this prespecified comparison directly evaluates the additive effect of anodal stimulation during LT. Fixed effects comprised group (two levels), time (modeled as a continuous variable representing measurement occasions coded as 0–4), day, and the group × time interaction. We prespecified the group × time interaction as the main test of interest. Time was modeled as a continuous variable to improve statistical efficiency and model stability given the limited sample size. This approach assumes a linear trend and may not fully capture nonlinear temporal dynamics.

We included baseline (day 1, pre-stimulation) values as covariates to adjust for initial differences. We also included age as a covariate to account for baseline imbalances and their potential influence on balance. We specified a random intercept for each participant to account for within-subject correlation. Given the modest sample size, we imposed no additional repeated covariance structures to ensure model stability and convergence. We fitted the models using restricted maximum likelihood and estimated degrees of freedom using the Satterthwaite approximation. Given the small sample size, this approach may be associated with instability in variance estimation; therefore, the results should be interpreted with caution.

For the primary outcome, comparisons involving the tDCS-only group were considered exploratory and were analyzed separately from the prespecified primary comparison. As a sensitivity analysis, we refitted the primary model treating time as a categorical rather than a continuous variable to avoid imposing a linear assumption on the temporal response pattern. This analysis was conducted to assess the robustness of the primary findings to model specification.

#### 2.15.2. Secondary Outcomes

For secondary outcomes, we applied LMMs to all three groups (sham tDCS + LT, anodal tDCS + LT, and anodal tDCS alone). Fixed effects included group (three levels), time (categorical), day, and the group × time interaction. For each outcome, we examined the main effects of group, time, and the group × time interaction.

When we identified a statistically significant effect (group, time, or group × time), we conducted post hoc comparisons using estimated marginal means. We adjusted pairwise comparisons for multiple testing using the Bonferroni correction. We included baseline values as covariates where appropriate. We calculated a random intercept for each participant.

### 2.16. Long-Term Effects (Days 1, 5, and 12)

To evaluate long-term effects, we analyzed outcomes assessed on days 1, 5, and 12 using separate LMMs, which included fixed effects for group (three levels), day (categorical: 1, 5, and 12), and the group × day interaction, with a random intercept for each participant.

For physical performance measures, we included only the pre-stimulation values (time = 0) on days 1, 5, and 12. We evaluated baseline-adjusted between-group differences on day 12 using model-based comparisons derived from estimated marginal means, with Bonferroni adjustment where applicable.

### 2.17. General Considerations

The LMM approach enabled inclusion of all available observations under the missing-at-random assumption without imputation. We performed all analyses using SPSS Statistics, version 29 (IBM Japan, Ltd., Tokyo, Japan). We used two-sided tests with a significance level of α = 0.05.

## 3. Results

### 3.1. Participant Characteristics

**Figure 2:**
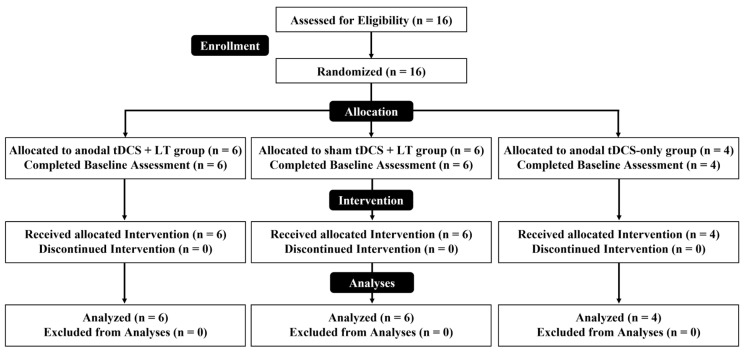
Flow chart of participants enrolled.

Sixteen participants met the inclusion criteria and were enrolled. Participants were randomly assigned to one of three intervention groups: anodal tDCS + LT (*n* = 6), sham tDCS + LT (*n* = 6), or tDCS only (*n* = 4). No dropouts or missing data occurred, and all participants were included in the analysis (Figure 2).

Baseline demographic and clinical characteristics are summarized in Table 1. The overall mean age of participants was 76.8 ± 4.9 years. A significant difference in age was observed among the three groups (*p* = 0.003). Post hoc Bonferroni comparisons revealed that the tDCS-only group was significantly younger than the sham + LT group (*p* = 0.008) and the tDCS + LT group (*p* = 0.005). Significant group differences were also observed in body weight (*p* = 0.028), body mass index (BMI; *p* = 0.007), and fat mass (*p* = 0.025). Post hoc analysis showed that body weight differed significantly between the tDCS + LT and tDCS-only groups (*p* = 0.031), BMI differed between the sham + LT and tDCS-only groups (*p* = 0.018), and fat mass differed between the sham + LT and tDCS-only groups (*p* = 0.042). No significant differences were observed among groups in sex distribution, height, fat-free mass, total body fat percentage, or total muscle mass. None of the participants met the diagnostic criteria for sarcopenia.

No statistically significant baseline differences were observed across intervention groups in physical performance measures, including bilateral handgrip strength, finger-pinch strength, knee extension strength, toe-pinch strength, stand-up test performance, two-step value, GLFS-25 score, LS severity, single-leg stance time with eyes open, and TUG test performance.

### 3.2. Within-Day Effects (Days 1–5)

#### 3.2.1. Primary Outcomes

An LMM adjusted for baseline values and age revealed a significant group × time interaction for right-sided single-leg stance time with eyes open between the tDCS + LT and sham + LT groups (F(1, 58) = 6.08, *p* = 0.017). The estimated interaction coefficient (β = 0.966) indicated a differential pattern of within-day change between groups, with the tDCS + LT group demonstrating greater improvements over time than the sham + LT group. In contrast, no significant interaction was observed on the left side (F(1, 58) = 0.53, *p* = 0.471).

As a sensitivity analysis, the primary outcome was reanalyzed using an LMM treating time as a categorical variable. In this analysis, the group × time interaction was not statistically significant (right side: *p* = 0.186; left side: *p* = 0.675).

**Figure 3:**
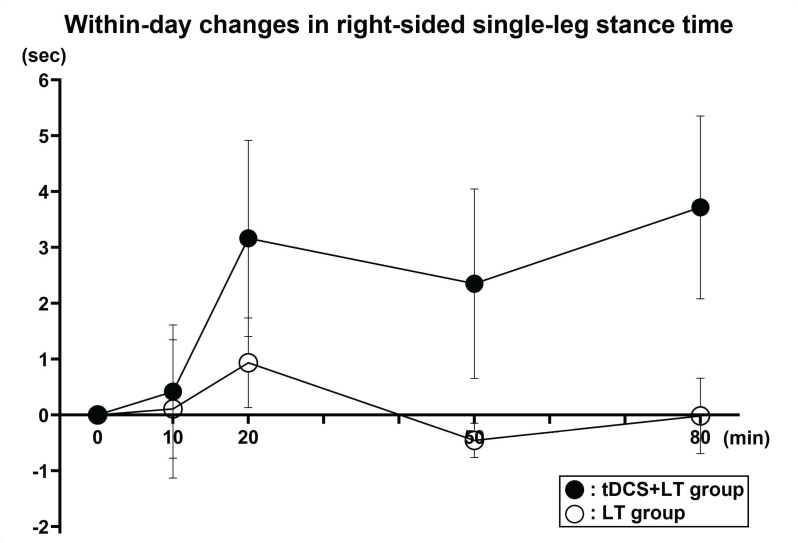
Within-day changes in right-sided single-leg stance time (normalized to baseline) in the tDCS + LT and LT groups.

Within-day absolute changes from baseline (Time 0) are shown in Figure 3 to facilitate visualization of this interaction. The sham + LT group exhibited minimal fluctuations around baseline across time points, whereas the tDCS + LT group demonstrated progressively greater increases in single-leg stance time, particularly at 20, 50, and 80 min after stimulation onset. These graphical trends are consistent with the significant group × time interaction observed in the mixed-effects model and illustrate a greater magnitude of within-day improvement in the combined intervention group.

Values represent mean absolute change (Δ seconds) from baseline (Time0) in right-sided single-leg stance time with eyes open. Error bars indicate standard errors of the mean calculated from individual change scores. Time points corresponded to pre-stimulation (Time0), 10 min (Time1), 20 min (Time2), 50 min (Time3), and 80 min (Time4) after stimulation onset. The tDCS-only group was not included because the primary analysis focused on the prespecified comparison between the tDCS + LT and LT groups to evaluate the additive effect of tDCS during locomotive training. tDCS + LT: anodal tDCS plus locomotive training, LT: sham tDCS plus locomotive training. The plotted values represent aggregated within-day changes averaged across days 1–5, consistent with the primary modeling approach.

#### 3.2.2. Secondary Outcomes

LMMs were used to examine within-day effects (days 1–5) on secondary outcomes (Table 2).

No significant group × time interactions were observed for handgrip strength (right or left), finger-pinch strength (right), knee extension strength (right or left), toe-pinch strength (right or left), stand-up test performance, or TUG test performance. However, a significant interaction was detected for the two-step value (F(8, 288) = 2.13, *p* = 0.033); post hoc Bonferroni comparisons did not reveal significant pairwise group differences.

Significant main effects of time were observed for finger-pinch strength on the left side (F(4, 220) = 3.06, *p* = 0.018), knee extension strength on the right side (F(4, 274) = 2.95, *p* = 0.021), toe-pinch strength on the right side (F(4, 277) = 6.87, *p* < 0.001), and TUG test performance (F(4, 266) = 8.46, *p* < 0.001). No significant time effects were observed for the remaining outcomes. Post hoc Bonferroni analyses indicated that right toe-pinch strength values at 20, 50, and 80 min were significantly greater than those at pre-stimulation (time 0). In addition, right toe-pinch strength at 10 min was significantly greater than that at 50 min. In the TUG test, performance at 20, 50, and 80 min was significantly faster than that at pre-stimulation. Additionally, performance at 10 min was significantly faster than that at 50 and 80 min. For finger-pinch strength (left) and knee extension strength (right), although significant time effects were observed, post hoc comparisons did not demonstrate consistent pairwise differences between specific time points. Overall, the secondary outcomes demonstrated time-dependent changes in several strength and mobility measures; however, these effects were largely independent of group allocation.

### 3.3. Long-Term Effects (Days 1, 5, and 12)

LMMs were used to evaluate long-term changes across days 1, 5, and 12 (Table 3).

No significant group × day interactions were observed for the LS stage, Loco-Check questionnaire score, GLFS-25 score, single-leg stance time (right or left), handgrip strength (right or left), finger-pinch strength (right or left), knee extension strength (right or left), toe-pinch strength (right or left), stand-up test performance, two-step value, or TUG performance.

Significant main effects of day were observed for right toe-pinch strength (F(2, 26) = 9.87, *p* < 0.001), left toe-pinch strength (F(2, 26) = 3.50, *p* = 0.045), and TUG performance (F(2, 26) = 34.51, *p* < 0.001). No other outcomes exhibited significant day effects. Post hoc Bonferroni analyses indicated that right toe-pinch strength on day 12 was significantly greater than that on day 1 (*p* < 0.001). In the TUG test, performance on days 5 and 12 was significantly improved compared with that on day 1 (both *p* < 0.001).

Overall, these findings suggest that certain lower extremity strength and mobility measures improved over time; however, these changes were not differentially influenced by group allocation.

### 3.4. Evaluation of Prespecified Hypotheses

Collectively, these findings partially support our prespecified hypotheses: the combined intervention enhanced the rate of within-day balance improvement (Hypothesis 1), and the additive effect was selectively expressed in postural control rather than isolated strength measures (Hypothesis 2), whereas sustained between-group differences were not observed, indicating limited support for cumulative long-term benefits under the present stimulation protocol (Hypothesis 3).

### 3.5. Safety

No serious adverse events were observed during the study period. None of the participants reported any discomfort, skin irritation, neurological symptoms, or other complications related to the tDCS or LT.

## 4. Discussion

This pilot randomized controlled trial examined whether anodal tDCS enhanced the effects of LT in older adults with LS. Three prespecified hypotheses were tested.

We hypothesized that combining anodal tDCS with LT would increase the rate of within-day improvement in balance performance. This hypothesis was supported by the significant group × time interaction for right-sided single-leg stance time and the steeper slope of improvement in the combined intervention group. Importantly, this interaction reflects an averaged within-session response pattern across all intervention days rather than distinct day-specific adaptations. Therefore, the present findings should be interpreted as indicating a generalized within-day facilitation effect, rather than evidence of consistent or cumulative changes across individual days.

Second, we hypothesized that the additive effect would be more pronounced in postural control measures than in isolated muscle strength measures. This hypothesis was also supported, as most strength-related outcomes lacked significant group-specific interactions, whereas the balance measure showed a clear differential response.

Third, we hypothesized that repeated sessions would produce cumulative functional benefits. However, no significant group × day interactions emerged in the long-term analyses, suggesting that although acute neuromodulation accelerated short-term adaptation, durable between-group differences were not achieved with the present five-session protocol.

Importantly, given the small sample size, these findings should be interpreted as preliminary and hypothesis-generating rather than confirmatory.

This study is among the first to investigate the additive effects of noninvasive cortical stimulation on balance performance in the context of LS. These findings suggest that central nervous system modulation selectively augments short-term postural adaptation when combined with task-specific training.

### 4.1. Primary Outcome: Single-Leg Stance Time with Eyes Open

#### 4.1.1. Interpretation of Lateral Asymmetry

Anodal stimulation of the primary motor cortex (M1) enhances cortical excitability [9]. Previous studies have reported that tDCS improves static and dynamic balance in inactive older adults [32,33]. However, bilateral M1 stimulation was applied in the present study, and lower limb dominance was not assessed. Therefore, the observed right-sided effects cannot be definitively attributed to hemispheric dominance. Without assessment of lower limb dominance, the interpretation of these lateralized findings remains speculative and should be interpreted with caution. Lateral asymmetry may reflect subtle baseline differences in neuromuscular control, side-specific responsiveness of postural circuits, or statistical variability inherent in modest sample sizes. Further studies incorporating limb dominance assessments and neurophysiological measures are required to determine whether this lateralized response represents a reproducible mechanistic effect or sampling variability. In addition, in a sensitivity analysis treating time as a categorical variable, the group × time interaction was not statistically significant. This finding suggests that the observed effect in the primary analysis may be influenced by the linear modeling assumption and highlights the importance of cautious interpretation. Accordingly, the present findings should be considered preliminary and potentially dependent on model specification.

#### 4.1.2. Magnitude of the Add-On Effect of Anodal tDCS

Because time was modeled as a continuous variable (coded 0–4), the interaction coefficient (β = 0.966) represents the additional linear rate of improvement per measurement interval in the combined intervention group relative to the LT-only group. Practically, for each successive within-day assessment, participants who received anodal tDCS plus LT improved by approximately 0.97 s more than those receiving LT alone.

Across the full within-day assessment window (Time 0 to Time 4), this corresponds to an estimated cumulative between-group difference of approximately 3–4 s in favor of the combined intervention group. Importantly, this reflects a widening difference in the trajectory of change between groups rather than a simple mean difference at a single time point.

In older adults, single-leg stance time is strongly associated with fall risk and future functional decline [29,34,35]. Incremental improvements of approximately 1 s per assessment interval may translate into clinically meaningful enhancements in postural stability when accumulated across repeated testing.

Notably, this interaction remained significant after adjusting for age. Given the established association between aging and impaired postural control [36,37], age is a potential confounder of balance-related outcomes. The persistence of the effect after age adjustment strengthens the interpretation that the additive benefit reflects neuromodulatory facilitation rather than demographic imbalance. In addition, the observed effects may reflect individual variability, which should be considered when interpreting results in a small sample.

#### 4.1.3. Implications for LS

Participants exhibited early-stage LS without sarcopenia, indicating preserved muscle mass but potential central or neuromotor constraints. Noninvasive cortical stimulation facilitates motor learning and skill acquisition [38,39]; these findings suggest that combining central neuromodulation with task-specific training may enhance short-term balance adaptation in this population.

Given the pilot design and small sample size, these results are hypothesis-generating and require confirmation in larger, adequately powered trials.

### 4.2. Secondary Outcomes

Most secondary outcomes showed no significant group × time interactions, indicating that the additive effect of anodal tDCS was not uniform across strength and functional measures. Although a significant interaction was observed for the two-step value, post hoc analyses revealed no consistent pairwise group differences; thus, this finding should be interpreted cautiously.

Several secondary measures exhibited significant time effects, including left finger-pinch strength, right knee extension strength, right toe-pinch strength, and TUG performance. These improvements occurred across groups and likely reflect practice effects, short-term motor adaptation, or repeated task engagement rather than stimulation-specific mechanisms [40,41,42]. Accordingly, they should not be interpreted as evidence of a specific neuromodulatory effect of tDCS. Rather, these findings support the notion that repeated task performance itself can enhance motor function in older adults.

Repeated exposure to functional tasks in a short timeframe may facilitate motor learning and enhance movement efficiency, particularly in older adults with preserved baseline function [43,44]. Similar short-term gains have been reported after repeated motor testing [45].

Importantly, the lack of consistent group-specific effects on strength measures suggests that acute neuromodulation preferentially affects neural mechanisms underlying postural control rather than directly enhancing isolated peripheral muscle strength [46]. Collectively, these secondary findings indicate that the additive neuromodulatory effect is most evident in balance-related performance, consistent with the primary outcome.

### 4.3. Long-Term Effects

Unlike the within-day findings, no significant group × day interactions were detected across days 1, 5, and 12. This indicates that the additive effect of anodal tDCS did not result in sustained between-group differences in this five-session protocol.

Nevertheless, significant day effects were observed for toe-pinch strength and TUG performance across groups. These align with evidence that structured exercise interventions improve lower extremity strength and mobility in older adults [47], and likely reflect training-related adaptations rather than persistent stimulation effects.

Anodal tDCS induces short-term increases in cortical excitability, but the effect’s durability depends on cumulative dose [48], repetition [49], and task engagement [50]. Five consecutive sessions may have facilitated acute motor adaptation without inducing consolidation-dependent plasticity detectable on day 12 [51,52].

Thus, although acute neuromodulation accelerates short-term balance adaptation, sustained improvements across days primarily reflect training effects in the current protocol. Future studies with longer durations or greater cumulative stimulation are needed to assess potential durable additive effects.

### 4.4. Limitations

This study has some limitations. First, the modest sample size, smaller than originally planned, may have limited statistical power and increased the risk of Type I and Type II errors. In particular, statistically significant findings in such a small sample may reflect either true effects or stochastic variability driven by individual responses. The small sample size also increases susceptibility to individual variability, which may disproportionately influence model estimates. Although no clear extreme outliers were visually identified, the possibility that individual responses influenced the observed effects cannot be excluded. Furthermore, statistical inference based on mixed-effects models in small samples may be unstable, even when using methods such as Satterthwaite’s approximation. Accordingly, more robust approaches, including small-sample bias-corrected variance estimators, may be warranted in future studies. Finally, the results of the primary analysis were sensitive to the modeling approach for time, as treating time as a categorical variable attenuated the statistical significance of the interaction. This suggests that the findings may depend on model specification and should be interpreted with caution. As a result of the limited sample size, stratified analyses by individual days were not performed, as this would have reduced statistical stability. Future studies with larger samples should incorporate day-specific analyses to evaluate potential learning or habituation effects across repeated sessions. Second, although randomization was performed, baseline differences in age and body composition were observed among the three groups. The primary analysis adjusted for age, and the main comparison focused on the LT and combined groups, between which age did not significantly differ; however, residual confounding cannot be completely excluded. Third, time was modeled as a linear continuous variable in the primary analysis. Although this approach enhances statistical power and model stability in small samples, it assumes a linear trajectory of change and may not fully capture potential nonlinear patterns of response over time. Fourth, bilateral M1 stimulation was applied, and lower limb dominance was not assessed. This limits the interpretation of lateralized findings, which should therefore be considered speculative. Consequently, the observed side-specific effects cannot be definitively attributed to hemispheric lateralization or dominance-related mechanisms. Fifth, the intervention consisted of five consecutive stimulation sessions. Although this may be sufficient to induce short-term modulation of motor performance, the dose may be inadequate to generate durable, consolidation-dependent plasticity. Longer intervention periods or higher cumulative stimulation doses may be required to examine sustained neuromodulatory effects. Sixth, the participants had early-stage LS without sarcopenia, reflecting relatively preserved baseline function. This may have introduced a ceiling effect in certain strength and mobility measures, potentially limiting detectable between-group differences. Seventh, no neurophysiological measurements (e.g., motor-evoked potentials or neuroimaging) were collected. Therefore, the mechanistic interpretation of cortical excitability and plasticity remains unclear.

## 5. Conclusions

In conclusion, anodal tDCS combined with LT was associated with greater within-day improvements in balance performance among older adults with LS. An additive effect was selectively observed in postural control in the primary analysis after adjusting for age. However, this effect was sensitive to the modeling approach for time, and no sustained between-group differences were detected over 12 days, suggesting that short-term neuromodulatory facilitation may not necessarily translate into durable functional gains under the five-session protocol. These findings should be interpreted as preliminary and hypothesis-generating. Nevertheless, they suggest a potential role for central neuromodulation as an adjunct to task-specific training and provide a rationale for future adequately powered studies to determine optimal stimulation parameters and long-term efficacy.

## Figures and Tables

**Table 1:** Baseline demographic and clinical characteristics of the participants.

Group	All (N = 16)		LT Group (N = 6)		tDCS + LT Group (N = 6)		tDCS Group (N = 4)		
	Mean	SD	Mean	SD	Mean	SD	Mean	SD	*p*-Values
Age	76.8	4.9	78.7	3.8	79.2	2.5	70.5	4.0	0.003 *
Sex (woman/man)	14/2		5/1		5/1		4/0		0.683
Height (cm)	149.6	7.3	150.7	9.8	150.3	4.8	147.1	7.8	0.742
Body composition									
Weight (kg)	51.0	8.0	49.4	9.6	57.0	4.1	44.4	1.7	0.028 *
BMI (kg/m^2^)	22.7	3.1	21.3	2.0	25.6	2.0	20.7	2.9	0.007 *
Fat mass (kg)	15.2	4.6	12.9	3.2	18.9	4.5	13.0	2.9	0.025 *
Fat-free mass (kg)	35.8	5.8	36.5	7.8	38.1	4.2	31.4	1.7	0.200
Total body fat (%)	29.5	6.1	26.2	4.2	33.1	6.6	29.3	5.9	0.138
Total muscle mass (kg)	33.9	5.5	34.6	7.3	36.0	4.1	29.8	1.6	0.211
Sarcopenia	0		0		0		0		—
Muscle strength (kgf)									
Handgrip (R)	20.8	4.7	20.2	5.4	22.0	5.7	19.8	1.2	0.743
Handgrip (L)	19.2	3.6	19.2	3.5	20.3	4.6	17.4	1.1	0.491
Finger pinch (R)	6.7	2.8	7.1	3.6	6.5	1.6	6.6	3.7	0.946
Finger pinch (L)	6.5	3.1	6.9	4.2	6.0	1.5	6.6	3.7	0.906
Knee extension (R)	19.8	5.7	18.9	8.1	22.1	2.1	17.8	5.3	0.472
Knee extension (L)	19.0	5.6	17.7	7.1	21.4	4.0	17.6	5.7	0.464
Toe pinch (R)	2.8	0.9	2.7	1.1	3.2	0.8	2.5	1.0	0.536
Toe pinch (L)	2.4	0.6	2.1	0.4	2.7	0.7	2.2	0.3	0.103
LS risk tests									
Stand-up test (Both legs 40 cm/30 cm/20 cm/ single leg 40 cm/30 cm/20 cm)	0/1/3/6/5/1		0/0/1/3/1/1		0/0/1/3/2/0		0/1/1/0/2/0		0.469
Two-step test value	1.4	0.1	1.3	0.1	1.4	0.1	1.4	0.2	0.594
GLFS-25 (points)	6.6	7.3	4.2	4.0	10.2	10.0	4.8	5.9	0.333
LS severity (LS stage: 0/1/2/3)	2/11/2/1		1/5/0/0		0/4/1/1		1/2/1/0		0.579
Single-leg standing with eyes open (R) (s)	20.6	10.9	21.3	13.3	16.5	9.5	25.5	9.1	0.467
Single-leg standing with eyes open (L) (s)	19.9	11.5	21.6	13.2	13.0	10.0	27.6	4.8	0.126
Timed Up-and-Go test (s)	6.5	0.8	6.3	0.9	6.3	0.4	7.1	0.9	0.222

Values are presented as mean ± standard deviation (SD) unless otherwise indicated. Categorical variables are presented as counts. *p*-values represent comparisons among the three groups (LT group, tDCS + LT group, and tDCS-only group) using one-way analysis of variance (ANOVA) for continuous variables and the chi-square test or Fisher’s exact test for categorical variables, as appropriate. Stand-up test values are presented as the number of participants who achieved each level in the following order: both legs 40 cm, both legs 30 cm, both legs 20 cm, single leg 40 cm, single leg 30 cm, and single leg 20 cm. Abbreviations: LT, locomotive training; tDCS, transcranial direct current stimulation; BMI, body mass index; GLFS-25, 25-question Geriatric Locomotive Function Scale; LS, locomotive syndrome. Linear mixed-effects models were used to test group × time interactions. Pairwise group comparisons were conducted for each outcome. F-values (df1, df2) and *p*-values are shown. (R): right side; (L): left side; LT: sham tDCS plus locomotion training; tDCS: anodal tDCS alone; and *: *p* < 0.05.

**Table 2:** Results of group × time interactions from linear mixed-effects models for secondary outcomes.

Secondary Outcomes	Effects	df1	df2	F-Value	*p*-Value
Handgrip strength (R)	Group	2	12	3.08	0.083
	Time	4	255	0.87	0.480
	Group × Time	8	255	1.01	0.426
Handgrip strength (L)	Group	2	12	3.51	0.063
	Time	4	257	0.62	0.648
	Group × Time	8	257	1.08	0.380
Finger-pinch strength (R)	Group	2	12	1.61	0.241
	Time	4	221	1.65	0.164
	Group × Time	8	221	1.06	0.396
Finger-pinch strength (L)	Group	2	12	2.87	0.096
	Time	4	220	3.06	0.018 *
	Group × Time	8	220	0.68	0.710
Knee extension strength (R)	Group	2	12	1.60	0.241
	Time	4	274	2.95	0.021 *
	Group × Time	8	274	0.54	0.822
Knee extension strength (L)	Group	2	12	0.56	0.588
	Time	4	282	1.86	0.117
	Group × Time	8	282	0.67	0.718
Toe-pinch strength (R)	Group	2	12	1.88	0.194
	Time	4	277	6.87	<0.001 *
	Group × Time	8	277	1.34	0.223
Toe-pinch strength (L)	Group	2	12	1.19	0.337
	Time	4	261	2.23	0.066
	Group × Time	8	261	0.48	0.868
Stand-up test	Group	2	12	0.32	0.730
	Time	4	283	0.58	0.674
	Group × Time	8	283	0.94	0.485
Two-step test value	Group	2	12	1.26	0.317
	Time	4	288	0.30	0.880
	Group × Time	8	288	2.13	0.033 *
Timed Up-and-Go test	Group	2	12	2.06	0.170
	Time	4	266	8.46	<0.001 *
	Group × Time	8	266	1.54	0.144

Linear mixed-effects models were used to test group × time interactions. Pairwise group comparisons were conducted for each outcome. F-values (df1, df2) and *p*-values are shown. (R): right side, (L): left side, and *: *p* < 0.05.

**Table 3:** Long-term effects (Days 1, 5, and 12) assessed using linear mixed-effects models.

	Effects	df1	df2	F-Value	*p*-Value
Locomotive syndrome stage	Group	2	13	1.27	0.314
	Day	2	26	1.629	0.216
	Group × Day	4	26	0.925	0.465
Loco-Check questionnaire	Group	2	13	0.304	0.743
	Day	2	26	2.072	0.146
	Group × Day	4	26	1.571	0.212
GLFS-25	Group	2	13	1.177	0.339
	Day	2	26	1.825	0.181
	Group × Day	4	26	0.615	0.655
Single-leg stance with eyes open (R)	Group	2	13	1.29	0.308
	Day	2	26	2.375	0.113
	Group × Day	4	26	0.646	0.635
Single-leg stance with eyes open (L)	Group	2	13	1.433	0.274
	Day	2	26	2.447	0.106
	Group × Day	4	26	1.17	0.347
Handgrip strength (R)	Group	2	13	1.80	0.204
	Day	2	26	0.38	0.686
	Group × Day	4	26	1.66	0.189
Handgrip strength (L)	Group	2	13	1.39	0.283
	Day	2	26	0.64	0.533
	Group × Day	4	26	0.89	0.482
Finger-pinch strength (R)	Group	2	13	0.23	0.802
	Day	2	26	1.43	0.257
	Group × Day	4	26	0.69	0.605
Finger-pinch strength (L)	Group	2	13	0.25	0.784
	Day	2	26	1.80	0.185
	Group × Day	4	26	0.84	0.510
Knee extension strength (R)	Group	2	13	3.46	0.063
	Day	2	26	2.78	0.081
	Group × Day	4	26	0.39	0.817
Knee extension strength (L)	Group	2	13	1.01	0.392
	Day	2	26	0.53	0.598
	Group × Day	4	26	0.10	0.982
Toe-pinch strength (R)	Group	2	13	1.31	0.302
	Day	2	26	9.87	<0.001 *
	Group × Day	4	26	1.44	0.250
Toe-pinch strength (L)	Group	2	13	1.27	0.313
	Day	2	26	3.50	0.045
	Group × Day	4	26	1.13	0.364
Stand-up test	Group	2	13	0.46	0.642
	Day	2	26	0.23	0.794
	Group × Day	4	26	0.66	0.625
Two-step test value	Group	2	13	0.16	0.856
	Day	2	26	1.37	0.272
	Group × Day	4	26	0.96	0.447
Timed Up-and-Go test	Group	2	13	2.58	0.114
	Day	2	26	34.51	<0.001 *
	Group × Day	4	26	0.78	0.548

Linear mixed-effects models were used to evaluate the effects of group (LT, tDCS + LT, and tDCS), day (Day 1, Day 5, and Day 12), and group × day interaction on long-term outcomes. F-values (df1, df2) and corresponding *p*-values are presented. Significant effects (*p* < 0.05) are indicated by an asterisk. Post hoc comparisons were conducted using Bonferroni correction when significant main effects were observed. Abbreviations: GLFS-25, 25-question Geriatric Locomotive Function Scale; LS, locomotive syndrome; (R), right side; (L), left side; LT, sham tDCS plus locomotive training; tDCS, anodal tDCS alone.

## Data Availability

The datasets generated during the current study are not publicly available because of participant privacy and ethical restrictions but are available from the corresponding author upon reasonable request.

## Abbreviations

The following abbreviations are used in this manuscript:

tDCS	transcranial direct current stimulation
LT	locomotive training
LS	locomotive syndrome
TUG	timed up-and-go
GLFS-25	25-question geriatric locomotive function scale
LMM	linear mixed-effects model
